# Modified Renshen Yangrong decoction enhances angiogenesis in ischemic stroke through promotion of MicroRNA‐210 expression by regulating the HIF/VEGF/Notch signaling pathway

**DOI:** 10.1002/brb3.2295

**Published:** 2021-08-01

**Authors:** Ce Liang, Teng Zhang, Xu‐Liang Shi, Lin Jia, Ya‐Li Wang, Cui‐Huan Yan

**Affiliations:** ^1^ Department of Hebei TCM Formula Granule Innovation Center Hebei University of Chinese Medicine Shijiazhuang China; ^2^ Department of Health Commission of Hebei Province Department of Health of Traditional Chinese Medicine Shijiazhuang China; ^3^ Department of Acupuncture and Moxibustion Hebei University of Chinese Medicine Shijiazhuang China; ^4^ Department of Respiratory Hebei Provincial Hospital of Traditional Chinese Medicine Shijiazhuang China; ^5^ Department of Teaching and Research Section of Integrative Medicine Hebei Medical University Shijiazhuang China; ^6^ Department of Internal Medicine Hebei University of Chinese Medicine Shijiazhuang China

**Keywords:** angiogenesis, HIF/VEGF/Notch, Ischemic stroke, miRNA‐210, Renshen Yangrong decoction

## Abstract

**Objective:**

This study aims to investigate the efficacy of modified Ginseng Yangrong decoction (GSYRD) promoting angiogenesis after ischemic stroke.

**Methods:**

In an in vivo study, rats that survived surgery were allocated into four groups: the control group and model group were treated with normal saline, the GSYRD group was treated with 18.9 mg/kg of GSYRD daily, and the positive control group was treated with Tongxinluo (TXL) (1 g/kg/d). At the end of the seven‐day treatment, the area of cerebral infarction, the expression changes of miRNA‐210 and ephrin A3 were determined. In an in vitro study, HUVECs were divided into a normal control serum group (NC group), normal control serum OGD group (Oxygen Glucose Deprivation group) (OGD group), OGD + drug‐containing serum group (OGD+GSYRD group), and OGD + drug‐containing serum + ES group (Endostatin group) (OGD+GSYRD+ES group). The cells in all groups except the NC group were cultured in a sugar‐free DMEM medium under hypoxia for 48 h. Cell proliferation, angiogenic structure formation ability, the expression changes of miRNA‐210, ephrin A3, and the HIF/VEGF/Notch signaling pathway‐related molecules were determined.

**Results:**

In vivo, GSYRD significantly reduced infarct size (*p* < .01), the expression of miRNA‐210 and ephrin A3 were decreased in the GSYRD group (*p* < .05). In vitro, the cell proliferation and tube formation ability were significantly increased in the GSYRD group (*p* < .05), and the expression of miRNA‐210 and ephrin A3 was decreased (*p* < .05). In addition, in the GSYRD group, the expression of the HIF/VEGF/Notch signaling pathway‐related molecules was significantly increased (*p* < .01 or *p* < .05).

**Conclusion:**

GSYRD promotes cerebral protection following angiogenesis and ischemic brain injury. The specific mechanism was activating the HIF/VEGF/Notch signaling pathway via miRNA‐210.

## Graphical abstract

This study aims to investigate the efficacy of modified Ginseng Yangrong decoction (GSYRD) promoting angiogenesis after ischemic stroke. GSYRD promotes cerebral protection following angiogenesis and ischemic brain injury. The specific mechanism was activating the HIF/VEGF/Notch signaling pathway via miRNA‐210.

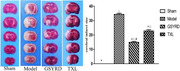

## 1 INTRODUCTION

Ischemic stroke is a common clinical disease with high mortality (D. Chen et al., 2015; Donnan et al., 2008; Lapergue et al., 2017; Rodgers, 2013). Interruption of cerebral artery blood flow and hypoxic‐ischemic necrosis of local brain tissue are the leading causes of ischemic stroke (Du et al., 2011). After the disease occurs, the blood flow in the ischemic center decreases rapidly, and some blood flow in the periphery penumbra still passes through. If the blood flow in this area is restored quickly, the functions of the patient could be improved (Sharp et al., 2000). The recovery of blood flow includes the opening of the infarcted blood vessels and the compensation of the collateral circulation. As a critical measure of early blood flow recovery, thrombolysis is often not practical due to strict time windows and indications. Therefore, promoting the regeneration of blood vessels and actively establishing the collateral circulation becomes an urgent and new treatment direction for ischemic stroke. Studies have shown that the establishment of collateral circulation can effectively reduce the area of cerebral infarction and improve the survival rate of nerve cells and prognosis (Bang et al., 2011; Kao et al., 2017; Souza et al., 2012).

Angiogenesis refers to the formation of new vascular networks by existing vascular endothelial cells by proliferation, migration, and budding and the delivery of nutrients and oxygen to various organs and tissues (Carmeliet, 2005). It is a crucial process of local blood supply (Ruan et al., 2015) and is regulated by various vascular growth factors. Studies have shown that miRNA regulation of tissue angiogenesis after ischemia has become one of the research hotspots at home and abroad. MicroRNAs‐210 are hypoxia‐specific microRNAs, which play an essential role in promoting angiogenesis by negatively regulating its target gene, ephrin A3 (Fasanaro et al., 2008). Ephrin A3 plays a vital role in the mechanism of VEGF (Vascular endothelial growth factor) signaling, promoting angiogenesis. HIF‐1 (HyPoxia‐inducible factor 1α) plays an important role as a core regulator of angiogenesis. When hypoxia occurs in tissues or cells, HIF‐1 binds specifically with VEGF and promotes the recovery of blood supply in ischemic areas by regulating the downstream Notch signaling pathway. Therefore, finding effective drugs that can promote angiogenesis, alleviate brain damage after ischemia and hypoxia, and understand the molecular mechanism of its occurrence is of great significance for the treatment of ischemic stroke.

Ginseng Yangrong decoction (GSYRD) is derived from “Taiping Huimin Hejiju Fang” and is composed of Radix Astragali, Radix Rehmanniae praeparata, Radix Angelicae sinensis, Radix Paeoniae Alba, Radix Codonopsis, Rhizoma Atractylodes alba, Poria, Radix Glycyrrhizae, Pericarpium Citri Reticulatae, Cortex Cinnamomi, Fructus Schisandrae, and Radix Polygalae (D. Chen., Lin, F., & Li, P. P., 2011). Modern pharmacological studies have shown that it can enhance the body's resistance to various diseases. British researchers explored the effects of GSYRD on normal human aortic endothelial cells through experimental research. The results showed that it could promote the proliferation of endothelial cells, inhibit the synthesis of endothelin, and accelerate its decomposition (Xuejiang et al., 2001). Our preliminary research results show that GSYRD can promote the proliferation and migration of HUVECs in a dose‐dependent manner (Zhu et al., 2017). However, the mechanical function of GSYRD is not fully grasped in promoting angiogenesis. Therefore, we investigated whether GSYRD can improve ischemia by promoting angiogenesis and whether the miRNA‐210 and HIF/VEGF/Notch signaling pathway is involved in the molecular mechanism of GSYRD.

## 2 MATERIALS AND METHODS

### 2.1 Experimental animals

In this trial, we selected adult male Sprague Dawley (SD) rats (250–280 g) purchased from Liaoning Changsheng Bio‐technology Co. (Liaoning, China) and kept in an animal room under standard conditions (temperature 22–25°C; humidity 60–70%). All animal care and experimental protocols complied with the Animal Management Rules of the Ministry of Health of the People's Republic of China and were approved by the Institutional Animal Care and Use Committee of the Hebei University of Chinese Medicine.

### 2.2 Establishment of the MCAO (Middle cerebral artery occlusion) model

Referring to the improved middle cerebral artery ischemia method in rats such as Longa, the specific steps were as follows: the SD rats were weighed, anesthetized with 20% uratan, and placed on the operating table in a supine position. The neck skin was cut, the neck fascia, platysma, sternocleidomastoid, and sternal hyoid muscles were bluntly separated. The right CCA (common carotid artery) bifurcation, internal carotid artery (ICA), and external carotid artery (ECA) were identified. The distal end of the ECA was ligated, and the proximal end of the heart was ligated for a short time. The artery clamp was completely clamped at 0.5 cm from the proximal end of the CCA bifurcation. The artery clamp completely clamped the ICA, and a “V” was cut along the distal end of the ECA. The ECA stump was straightened along the ICA, a 0.2 mm diameter blunt nylon thread was inserted into the artery, the ICA was passed through the CCA bifurcation (releasing the arterial clip), and the beginning of the middle cerebral artery was entered to block the blood flow of the middle cerebral artery. The silk thread was slowly pushed until stopped by resistance, the ECA line was ligated, the CCA artery clamp was relaxed for hemostasis, the skin was sutured, and the rat was placed inside a cage to have free access to food and water. Rats were given an incandescent lamp (100 W) during the operation and before waking, maintaining an anal temperature of about 36°C and room temperature of 25°C. Animals operated in the control group only had blood vessels separated and exposed without any damage.

### 2.3 Drug treatment

GSYRD was purchased from the National Medical Hall of Hebei College of Traditional Chinese Medicine (Shijiazhuang, China). The solution was concentrated into a mixture containing 2 g/ml of the crude drug. ES is a specific inhibitor of HIF‐VEGF available from PeproTech (UK). Tongxinluo ultrafine powder was purchased from Yiling Pharmaceutical Co., Ltd. in Shijiazhuang and dissolved in DMEM medium. The solution was adjusted to a final concentration of 2 mg/ml and preserved at −20°C for subsequent use.

The rats that survived the surgery were allocated into four groups: the control group (*n* = 18), the model group (*n* = 18), the GSYRD group (18.9 mg/kg/d, *n* = 18), and the TXL group (1 g/kg/d, *n* = 18). The GSYRD and TXL groups were intragastrically administrated for 7 d; an equal amount of normal saline was intragastrically administrated every day in the model and control groups.

### 2.4 Preparation of drug‐containing serum

Healthy adult male SD rats weighing 250–280 g were randomly divided into two groups: the normal control group and the GSYRD group. The GSYRD group was administered 18.9 g/kg twice a day for three days. One hour after the last administration, the rats were routinely anesthetized. Blood was taken from the abdominal aorta, left at room temperature for 2 h, centrifuged for 20 min. The serum was collected, inactivated at 56°C for 30 min, filtered through a 0.22 μm filter, and stored at −80°C. The normal control group was given the same amount of normal saline, and the other groups were prepared with the medicinal serum of the GSYRD.

### 2.5 Cell culture and treatment

Human umbilical vein endothelial cells (HUVECs) were cultured in DMEM (Gibco, USA), supplemented with 10% fetal bovine serum (Gibco, USA), 100 U/ml penicillin, and 100 g/ml streptomycin. The HUVECs were cultured in humidified air with 5% CO_2_ at 37°C. The whole experimental process conformed to the principles outlined in the Declaration of Helsinki. The cells were divided into the normal control serum group (NC group), the normal control serum OGD group (OGD group), the OGD + drug‐containing serum group (OGD+GSYRD group), and the OGD + drug‐containing serum + ES group (OGD+GSYRD+ES group). The cells in all groups except the NC group were cultured in a sugar‐free DMEM medium under hypoxia for 48 h. The hypoxic culture conditions were 1% O_2_, 94% N_2_, and 5% CO_2_.

### 2.6 The calculation of the infarct area

After treatment, six animals from each group were sacrificed, and the whole brain was excised immediately and then stored at −20°C for approximately 20 min after PBS washing to harden tissue. Then, the brain was cut into five pieces (each piece is about 2 mm thick), incubated with 2% TTC, covered with foil, and incubated in a 37°C water bath for 30 min. The brain slices were intermittently turned over to ensure the section fully contacted the dye solution. Sections were photographed, and the size of the infarcted region was evaluated. The non‐ischemic area was red, while the infarcted tissue was white. All sections were fixed in 4% paraformaldehyde, and the area of infarct was calculated with ImageJ.

### 2.7 Immunofluorescence staining

The SD rats in each group were sacrificed, and the brain tissue of the ischemic cortex was taken and fixed with 4% paraformaldehyde for 6 h. It was then embedded in paraffin. The paraffin was cut into 5‐micron slices and transferred to the slide. Tissue sections were incubated overnight with anti‐ephrin A3 antibody (1:200), washed four times with PBS, then incubated with goat anti‐rabbit secondary antibody (1:200) for 1 h, and washed four times with PBS, incubated with DAPI for 1 min, rinsed with PBS and finally sealed with a coverslip. All images were captured on a fluorescence microscope (OLUMPUS, Japan).

### 2.8 Quantitative real‐time PCR

TriPure Reagent (Takara Bio Inc., Dalian, China) was used for isolating total RNA from ventricular tissue. The tissue was directly lysed by mixing with 1 ml of TriPure Reagent and homogenized using a homogenizer. Then 200 μl of chloroform was added to the homogenized sample and incubated for 25 min at room temperature. Subsequently, RNA was precipitated by mixing with isopropyl alcohol. An ultraviolet spectrophotometer was used to measure the absorbance of RNA at 260 and 280 nm, and the concentration of RNA was determined. Then mRNA was isolated from total RNA using Oligo (dT), and reverse transcribed into first‐strand complement DNA (cDNA) and amplified using an SYBR GREEN Master Mix cDNA synthesis kit. The reaction system included 1 μl of cDNA, 10 μl of 2 × SYBR GREEN Master Mix, and 0.5 μl of each primer. The PCR condition was as follows: preincubation at 94°C for 2 min, followed by 40 cycles of denaturation at 94°C for 15 s, and annealing/extension at 60°C for 30 s using Exicycler™ 96 detection system. The primers are listed in Table 1.

**TABLE 1:** The primers of the mRNA

Items	Primers
Ephrin A3	Forward 5′‐ GAGGGTGGGAGGAAGTAAGC −3′
	Reverse 5′‐ TGTCGTTGTCCGATTCATAG −3′
r‐miRNA‐210	Forward 5′‐ GCGAGCCACTGCCCACAG −3′
	Reverse 5′‐ GTGCAGGGTCCGAGGTATTC −3′
h‐miRNA‐210	Forward 5′‐ GCCGAGCCCCTGCCCACCGC −3′
	Reverse 5′‐ GTGCAGGGTCCGAGGTATTC −3′
HIF‐1α	Forward 5′‐ GCAGGAAAAGGAGTCATAGA −3′
	Reverse 5′‐TAGTAGCTGCATGATCGTCT −3′
VEGF	Forward 5′‐TCACCAAGGCCAGCACATAG −3′
	Reverse 5′‐GGGCACCAACGTACACGC −3′
VEGFR2	Forward 5′‐AGGTGACTGAGTGCAGCGAT −3′
	Reverse 5′‐TAGACATAAATGACCGAGGC −3′
DLL4	Forward 5′‐AAACCAGCACCCTCACAAGG −3′
	Reverse 5′‐TGACAGCCCGAAAGACAGAT −3′
Notch1	Forward 5′‐AACAGCGAGGAAGAGGAGGA −3′
	Reverse 5′‐GCATCAGAGCGTGAGTAGCG −3′
r‐b‐actin	Forward 5′‐ GGAGATTACTGCCCTGGCTCCTAGC −3′
	Reverse 5′‐ GGCCGGACTCATCGTACTCCTGCTT −3′
h‐b‐actin	Forward 5′‐GGCACCCAGCACAATGAA −3′
	Reverse 5′‐TAGAAGCATTTGCGGTGG −3′
r‐U6	Forward 5′‐ CAAATTCGTGAAGCGTTCCATA −3′
	Reverse 5′‐ GTGCAGGGTCCGAGGTATTC −3′
h‐U6	Forward 5′‐ GCTTCGGCAGCACATATACT −3′
	Reverse 5′‐ GTGCAGGGTCCGAGGTATTC −3′

### 2.9 MTT assay

MTT Assay Cell viability was determined using the 3‐(4,5 dimethylthiazol‐2‐yl)‐2,5‐diphenyltetrazolium bromide (MTT) assay. Endothelial cells were cultured in a 96‐well plate and cultured under the above conditions. The cells were incubated for 4 h in a medium containing 0.5% MTT, the yellow mitochondrial dye. The reaction was terminated by adding 150 μl DMSO to the cells and incubating them for 10 min. Absorbance at 570 nm was recorded with an enzyme‐linked immunosorbent assay plate reader.

### 2.10 Tube formation assay

Tube formation assays were performed on Matrigel (BD Biosciences, USA) to assess the effect of AS‐VI on the HUVECs’ morphogenesis and tube formation ability. The HUVECs in the logarithmic growth phase were trypsinized, resuspended in the DMSO medium, and plated on a 96‐well plate coated with Matrigel, then incubated for 24 h in a 37°C incubator. The formation of the tube‐like structure was observed under an inverted phase‐contrast microscope, and the number of lumens formed under five fields of view was randomly calculated.

### 2.11 Flow cytometry

Cells in each group were collected and digested with trypsin, then centrifuged for 5 min; the antibody was added in the dark. The cells were centrifuged and the supernatant discarded. Each cell sample had 500 μl of the staining buffer added for 15 min, after which flow cytometry was performed.

### 2.12 Western blotting

The total protein was isolated from HUVECs according to the standard protocols. Subsequently, the protein concentration was determined by BCA assay (Wanleibio Co., Ltd., China). The separated proteins were then transferred onto polyvinylidene fluoride (PVDF) membranes with a transfer box. The resulting membranes were blocked with 5% skim milk overnight and incubated at 4°C and then incubated with corresponding primary antibodies obtained from Wanleibio (Shenyang, China), including anti‐HIF‐1α (1:500), anti‐VEGF (1:500), anti‐VEGFR2 (1:500), anti‐DLL‐4 (1:500), anti‐Notch1 (1:1000), anti‐β‐actin (1:1000), overnight at 4°C. After carefully washing with tris‐buffered saline with Tween 20 (TBST), the membrane was incubated with HRP‐conjugated secondary antibodies (1:5000) at 37°C for 45 min. The membranes were developed using the Super Ecl Plus sensitization solution, and the gels were exposed to a scan. The mean gray values of the bands were analyzed by ImageJ.

### 2.13 Statistical analysis

We used the software program SPSS 22.0 (IBM, Armonk, NY, USA) to conduct the statistical analysis. Continuous variables were expressed as mean ± SD. Discontinuous variables were expressed as a percentage (%). For multiple comparisons, each value was compared by one‐way ANOVA following the Dunnett test when each datum conformed to a normal distribution, while the non‐normally distributed continuous data were compared using non‐parametric tests. The counting data were tested by the chi‐square test. A value of *p* < .05 was considered statistically significant.

## 3 RESULTS

### 3.1 GSYRD reduces infarct size in rats with cerebral ischemia

**FIGURE 1:**
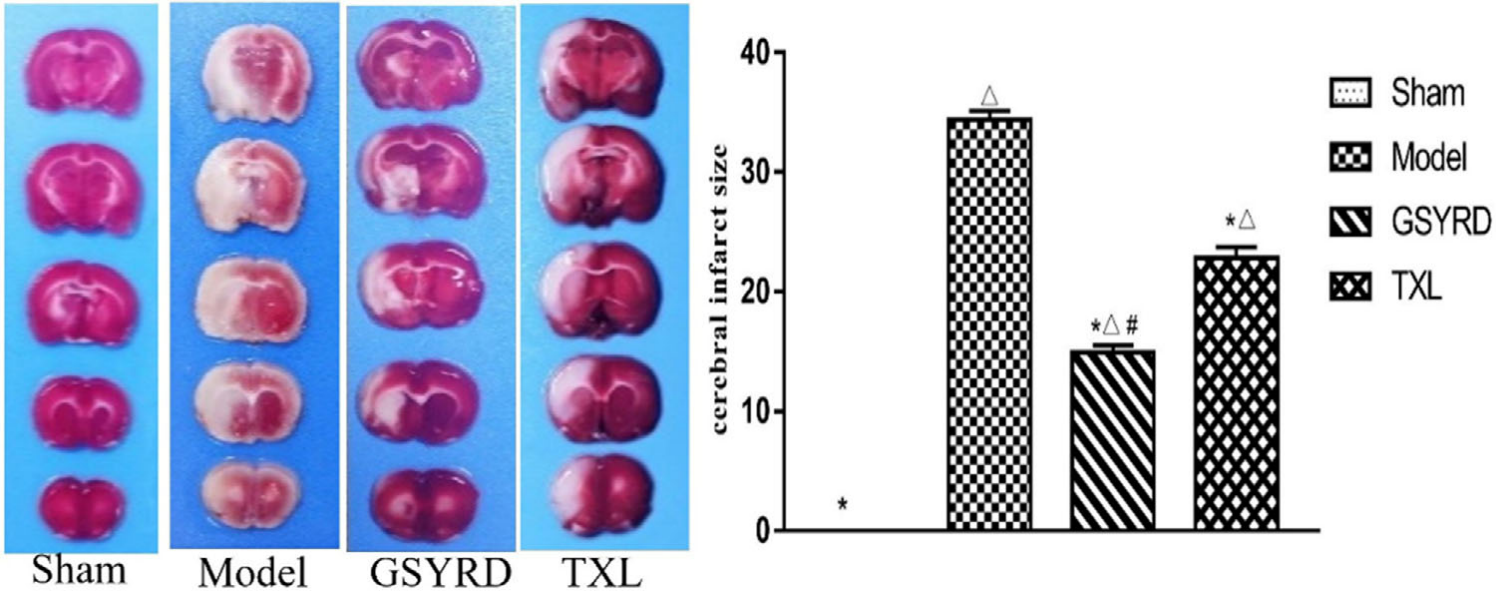
The protective effect of GSYRD on cerebral ischemia in rats. (A) Representative photos of TTC stained brain slices. The white region shows the infarct tissue, and the red region shows the viable tissue. (B) statistical data of infarct size. All data are expressed as mean ± SD. ^△^ *p* < .01 vs. sham; ^*^ *p* < .01 vs. model, ^＃^ *p* < .05 vs. TXL

TTC staining showed that infarct size was lower in the GSYRD and TXL groups compared with the model group, and the infarct size of the GSYRD group was smaller than the TXL group (Figure 1A,B). It shows that the treatment of the GSYRD group has a better therapeutic effect.

### 3.2 Changes of miRNA‐210 and ephrin A3 after GSYRD administration

**FIGURE 2:**
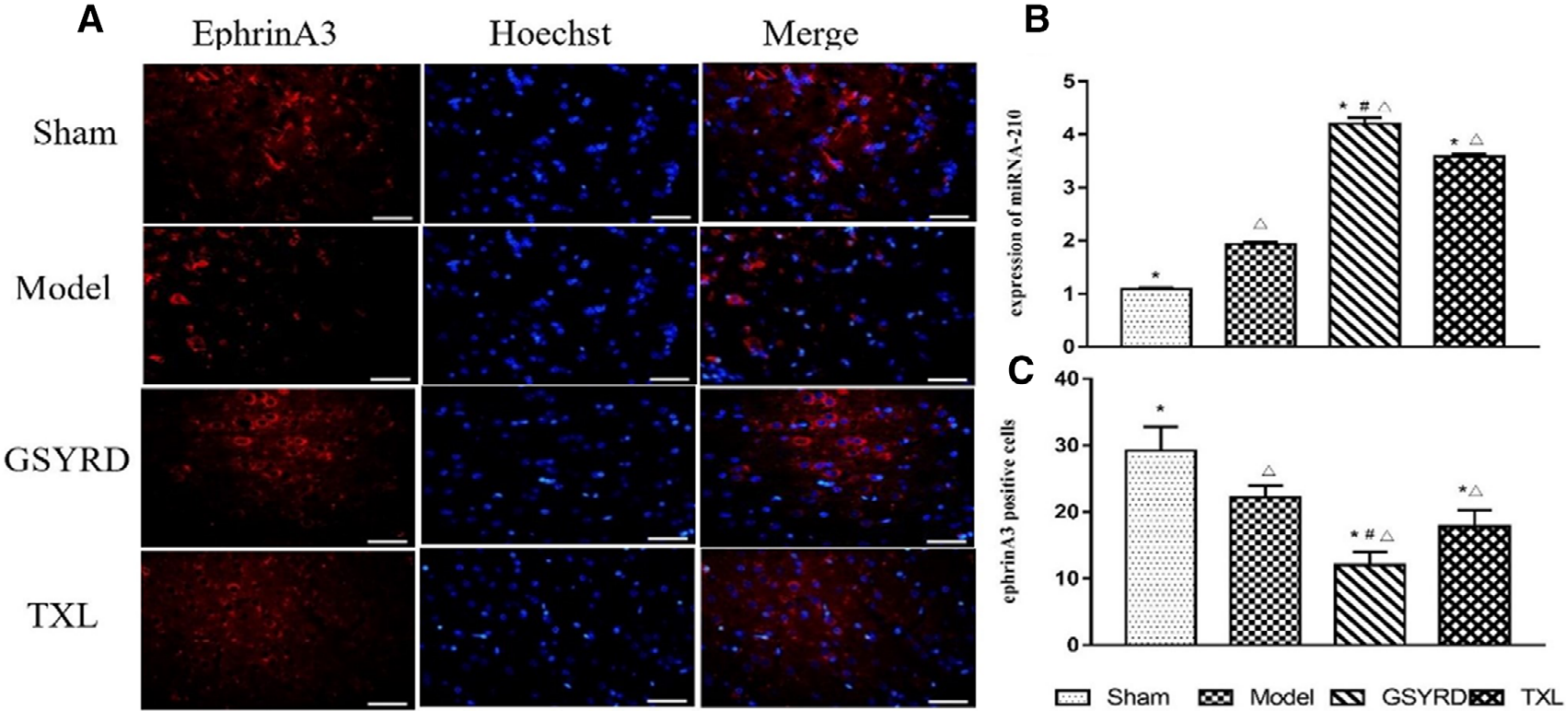
The changes of Ephrin A3 and miRNA‐210 expression after treated with GSYRD in rats. (A) Representative images of Ephrin A3. (B) Level of miRNA‐210 in rats from different groups measured by RT‐qPCR. (C) Fluorescence intensity of Ephrin A3. Data are expressed as mean ± SD, ^△^ *p* < .01 vs. sham; ^*^ *p* < .01 vs. model, ^＃^ *p* < .05 vs. TXL

After cerebral ischemia, the expression of miRNA‐210 and its target gene, ephrin A3, will change accordingly. RT‐qPCR detected the expression of miRNA‐210, and the change of ephrin A3 was observed by immunofluorescence staining. Compared with the control group, the expression of the model, the GSYRD, and the TXL groups miRNA‐210 was increased (Figure 2B), and the expression of ephrin A3 was decreased (Figure 2A,C). Among them, the expression of miRNA‐210 in the GSYRD group was higher than in the model and TXL groups (Figure 2B), and the expression of ephrin A3 was lower than in the model and TXL groups (Figure 2A,C). It indicates that GSYRD has a regulatory effect on miRNA‐210 and ephrin A3.

### 3.3 GSYRD augmented proliferation and tube formation in HUVECs

**FIGURE 3:**
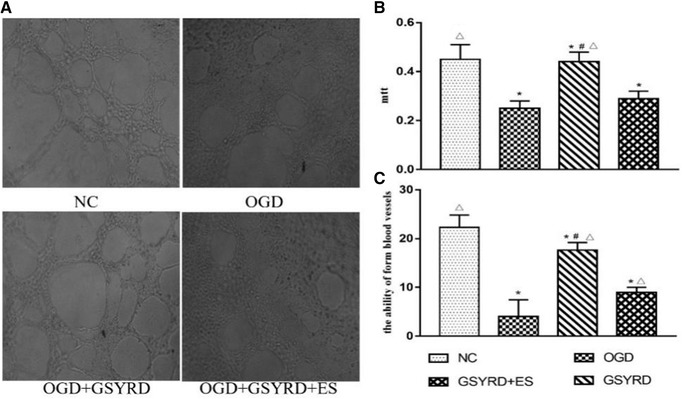
GSYRD promoted proliferation and tube formation of HUVECs. (A) Representative images on the formation of tubes. (B) Statistical analysis of proliferation. (C) Statistical analysis of tube formation. Data are expressed as mean ± SD, ^*^ *p* < .01 vs. NC; ^△^ *p* < .01 vs. OGD, ^＃^ *p* < .05 vs. OGD+GSYRD+ES

To explore the roles of the pro‐angiogenesis effect induced by GSYRD, we investigated cell proliferation and tube formation in GSYRD treated HUVECs using MTT and Matrigel tube formation assays, respectively. As shown in Figure 3, compared with the OGD group, the GSYRD group has a significantly increased cell proliferation ability and can effectively form a slender capillary‐like structure. After the addition of ES, the cell's proliferative capacity and angiogenic ability were significantly inhibited.

### 3.4 GSYRD stimulates angiogenesis through the HIF/VEGF/Notch pathway

**FIGURE 4:**
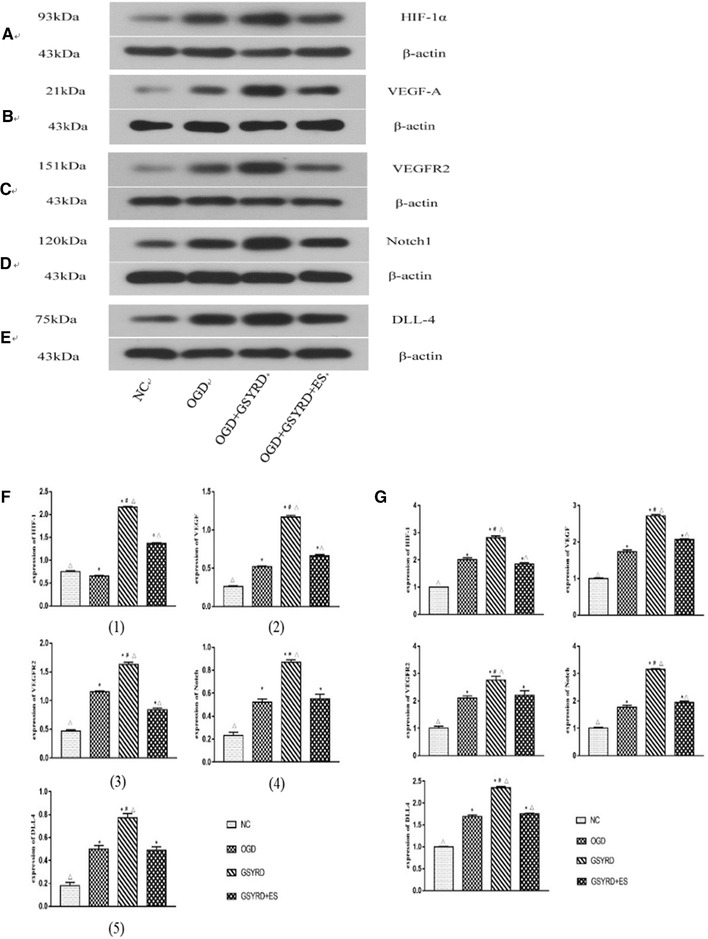
Assessment of signaling pathway activation after GSYRD treatment. (A–E) Expression of HIF/VEGF/Notch signaling pathway from different groups measured by western blotting; the grouping from left to right is: NC, OGD, GSYRD, OGD+GSYRD, OGD+GSYRD+ES. (F) Quantified western blotting results of HIF‐1α, VEGF, VEGFR2, Notch, DLL4 are provided, and panel (f 1) represents western blot analysis shown in (A), panel (f 2) represents western blot analysis shown in (B), panel (f 3) represents western blot analysis shown in (C), panel (f 4) represents western blot analysis shown in (D), panel (f 5) represents western blot analysis shown in (E). (G) Level of these pathway factors measured by RT‐qPCR. Data are expressed as mean ± SD, ^*^ *p* < .01 vs. NC; ^△^ *p* < .01 vs. OGD, ^＃^ *p* < .05 vs. OGD+ GSYRD +ES

The HIF/VEGF/Notch pathway has been shown to play an important role in the angiogenesis process. To clarify the effect of GSYRD on the HIF/VEGF/Notch pathway, we introduced inhibitor ES into cells. The introduction of ES reduced the expression of the HIF/VEGF/Notch pathway‐related molecular proteins and mRNA, and the expression of each protein and mRNA of the GSYRD group was higher than the OGD group, which explains that the addition of ES weakens the role of GSYRD (Figure 4). These results indicate that GSYRD can activate the HIF/VEGF/Notch pathway.

### 3.5 GSYRD regulated miRNA‐210 and ephrin A3 expressions

**FIGURE 5:**
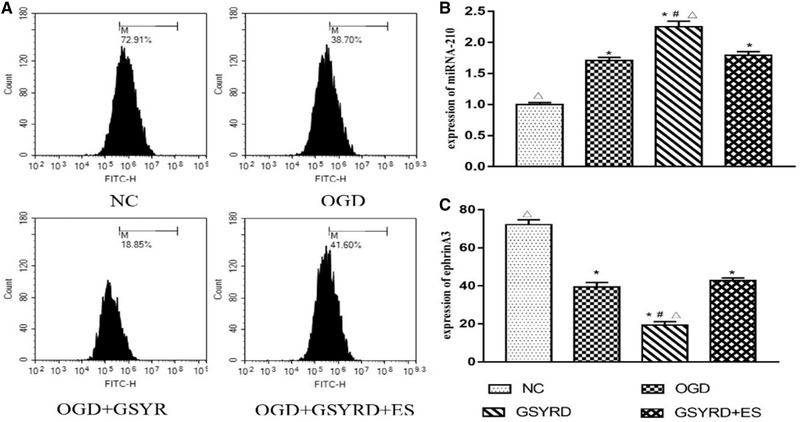
The changes of Ephrin A3 and miRNA‐210 expression after treated with GSYRD in HUVECs. (A) Representative images of Ephrin A3. (B) Level of miRNA‐210 in cells from different groups measured by RT‐qPCR. (C) Statistical analysis of Ephrin A3. Data are expressed as mean ± SD, ^*^ *p* < .01 vs. NC; ^△^ *p* < .01 vs. OGD, ^＃^ *p* < .05 vs. OGD+ GSYRD +ES

To further verify the effect of GSYRD on miRNA‐210, changes in miRNA‐210 and ephrin A3 in GSYRD treated cells were measured using RT‐qPCR and flow cytometry, respectively. The results showed that compared with the OGD group, the expression of miRNA‐210 in the GSYRD group was significantly increased (Figure 5B), and the expression of ephrin A3 decreased considerably (Figure 5A,B). With the addition of ES, the expression of miRNA‐210 and ephrin A3 was affected. This indicates that GSYRD can indirectly regulate the expression of miRNA‐210 and ephrin A3 in cells.

## 4 DISCUSSION

The major findings from this study are that GSYRD can promote the expression of miRNA‐210, inhibit the expression of ephrin A3, and reduce the infarct size in vivo. In vitro studies have shown that GSYRD can significantly increase cell viability, promote cell proliferation, enhance angiogenesis, regulate the expression of miRNA‐210, inhibit the expression of ephrin A3, and promote angiogenesis of HUVECs cells after hypoxia. Furthermore, the HIF/VEGF/Notch signaling pathway that took a critical part in GSYRD‐induced angiogenesis was identified.

HUVECs have become an essential source of vascular endothelial cells in vitro, which retains the characteristics of vascular endothelial cells, such as the expression of endothelial cell markers vWF factor, CD31, growth factor receptor, cytokines, and other vascular ligands, such as VEGF, and ANG‐n. It has the potential of stem cells. Vascular endothelial progenitor cells (EPCs), the precursor cells of vascular endothelial cells, are derived from umbilical vein blood and cultured in HUVECs, which are more in line with human physiological characteristics than animal endothelial cells. It is beneficial to observe the changes of biologically active substances in human endothelial cells after hypoxia and the corresponding pathological changes of organs and tissues after hypoxia and its mechanism of action. Many experiments selected HUVECs as the research object instead of directly using the arterial and venous endothelial cells. The main reason is that the latter two are differentiated cells and cannot have the ability to propagate infinitely. The HUVECs can be passaged multiple times for easy experimental operation. Therefore, we also selected HUVECs as the research object.

The study has shown that under the guidance of traditional Chinese medicine theory, according to the symptoms and characteristics of patients, the Chinese herbal compound is intended to contain a combination of various plants and minerals, which can improve clinical efficacy (Liu et al., 2007). It is believed that, at least in certain formulations, multiple components can bind to multiple targets to exert a synergistic therapeutic effect. The synergism between component herbs can play an essential role in that the effects of a combination of two herbs can be significantly greater than that of either in isolation (Liu et al., 2007; Wang et al., 2008; Xuejiang et al., 2001; Zhu et al., 2017). GSYRD is a traditional ancient prescription of Chinese medicine for qi‐supplementing, blood‐nourishing, Xin‐nourishing, and mind‐tranquilizing (Uehiyama, 1994). Modern studies show that it affects aging, enhances immunity, and improves anemia, and is frequently used to treat various diseases, including malignant tumors (Y. Z. Chen, Lin, F., Zhuang, G. B., et al., 2011).

HIF‐1α is a specific marker of hypoxia, a key regulator upstream of the VEGF signaling pathway, and a critical transcription factor that triggers the expression of various hypoxic stress proteins (Zhou et al., 2008). VEGF is an important factor regulating the promotion of angiogenesis, promoting the division and proliferation of vascular endothelial cells, and finally inducing the formation of new blood vessels (Ferrara & Davis‐Smyth, 1997; Hicklin & Ellis, 2005). Moreover, it plays a key role in neovascular remodeling after ischemic stroke (Ma et al., 2012). The Notch signaling pathway is involved in forming the collateral network of ischemic stroke, and inhibition of the Notch signaling pathway can impair the repair of post‐ischemic angiogenesis (Cristofaro et al., 2013). The expression of HIF‐1α is significantly increased after hypoxia, which promotes the expression of VEGF, activates the Notch signaling pathway and transcription of downstream target genes, promotes cell proliferation, survival, vascular endothelial cell migration, and arteriovenous differentiation.

To verify whether GSYRD promotes angiogenesis through the HIF/VEGF/Notch signaling pathway, we used endostatin (ES), a signal transduction inhibitor of VEGF. We observed the level of the HIF/VEGF/Notch protein and molecular changes by western blot and RT‐qPCR method and found that the expression level of related factors was significantly increased after the addition of GSYRD. When we added ES, the HIF/VEGF/Notch signaling pathway was blocked, and the pro‐angiogenic function of GSYRD was greatly inhibited, even lower than the control group. The above experimental results indicate that GSYRD promotes angiogenesis by activating the HIF/VEGF/Notch signaling pathway.

MiRNAs are widely involved in various physiological and pathological metabolic processes in vivo, especially in angiogenesis under hypoxia (Chan et al., 2012). Among them, miRNA‐210 is a hypoxia‐specific miRNA, which can be stably expressed in different concentrations of hypoxic environments and different cell types. In hypoxia, miRNA‐210 promotes endothelial cells to form blood vessels by inhibiting the expression of the target gene ephrin A3 (Ivan et al., 2008).

Liang et al. (2020) have reported that miRNA‐210 can regulate the expression of the HIF/VEGF/Notch pathway‐associated molecules and participate in the process of promoting angiogenesis after hypoxia. In previous research, the expression of VEGF is regulated by many miRNAs, and it is predicted by computer software that miRNA‐210 can regulate VEGF, which affects the process of angiogenesis (Hua et al., 2006). miRNA‐210 can regulate the expression of ephrin A3, promote the differentiation of HUVECs into capillary‐like structures, and promote the migration of HUVECs under the action of VEGF (Huang et al., 2010). Simultaneously, miRNA‐210 can promote angiogenesis in normal brain tissue by up‐regulating VEGF expression (Zeng et al., 2014). Kati et al (Pulkkinen et al., 2008) and other studies found that miRNA‐210 expression was up‐regulated in vitro and in vivo under hypoxic conditions, HIF‐1α activity was increased, and miRNA‐210 expression was induced, indicating that the HIF‐1α signal transduction pathway was involved in the transcription of miRNA‐210. miRNA‐210 can activate the expression of Notch1 signaling molecules in the ischemic cortex of adult rats. The expression of both is significantly up‐regulated, and overexpression miR‐210 caused up‐regulation of Notch signaling molecules and induced endothelial cells to migrate and form capillary‐like structures (Lou et al., 2012). Therefore, based on the enhanced expression of miRNA‐210 and the HIF/VEGF/Notch pathways, we found that the expression of miRNA‐210 and the HIF/VEGF/Notch pathways were consistent after GSYRD addition, showing an upward trend, which confirmed the mechanism of AS‐IV activating the HIF/VEGF/Notch signaling pathway to promote angiogenesis through miRNA‐210.

There were some limitations in this study. The specific active ingredients of GSYRD still need further research.

## 5 CONCLUSION

GSYRD promotes cerebral protection following angiogenesis and ischemic brain injury. The specific mechanism was activating the HIF/VEGF/Notch signaling pathway via miRNA‐210. GSYRD has good therapeutic ability as a treatment of ischemic diseases.

## CONFLICT OF INTEREST

Authors declare no conflicts of interest.

## AUTHOR CONTRIBUTIONS

Ce Liang, Teng Zhang, Ya‐Li Wang and Cui‐Huan Yan were involved in drafting the manuscript and revising it critically for important intellectual content and conception and design; Xu‐Liang Shi made substantial contributions to acquisition and interpretation of data; Lin Jia analyzed the data; All authors gave final approval of the version to be published.

## Data Availability

The datasets generated and/or analyzed during the current study are not publicly available due to the lack of an online platform but are available from the corresponding author on reasonable request.
