# Correction

**DOI:** 10.1002/brb3.3242

**Published:** 2023-08-29

Liang, C., Zhang, T., Shi, X.‐L., Jia, L., Wang, Y.‐L., & Yan, C.‐H. (2021). Modified Renshen Yangrong decoction enhances angiogenesis in ischemic stroke through promotion of MicroRNA‐210 expression by regulating the HIF/VEGF/Notch signaling pathway. *Brain and Behavior, 11*, e2295.

On page 6, in Figure 2A of Results section of article Liang et al. (2021), the Model group “Hoechst” picture is incorrect, and this should be the picture given below.

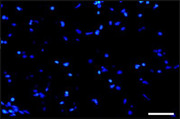

We apologize for this error.

